# Ketamine Effects on Memory Reconsolidation Favor a Learning Model of Delusions

**DOI:** 10.1371/journal.pone.0065088

**Published:** 2013-06-12

**Authors:** Philip R. Corlett, Victoria Cambridge, Jennifer M. Gardner, Jennifer S. Piggot, Danielle C. Turner, Jessica C. Everitt, Fernando Sergio Arana, Hannah L. Morgan, Amy L. Milton, Jonathan L. Lee, Michael R. F. Aitken, Anthony Dickinson, Barry J. Everitt, Anthony R. Absalom, Ram Adapa, Naresh Subramanian, Jane R. Taylor, John H. Krystal, Paul C. Fletcher

**Affiliations:** 1 Department of Psychiatry, Ribicoff Research Facility, Yale University, New Haven, Connecticut, United States of America; 2 Brain Mapping Unit, Department of Psychiatry, University of Cambridge, School of Clinical Medicine, Addenbrooke's Hospital, Cambridge, United Kingdom; 3 University of Cambridge, School of Clinical Medicine, Addenbrooke's Hospital, Cambridge, United Kingdom; 4 Department of Psychology, University of Cambridge, Cambridge, United Kingdom; 5 University of Birmingham, School of Psychology, Edgbaston, Birmingham, United Kingdom; 6 Department of Anesthesiology, University Medical Center Groningen, Groningen University, Groningen, The Netherlands; 7 Division of Anaesthesia, School of Clinical Medicine, University of Cambridge, Addenbrooke's Hospital, Cambridge, United Kingdom; 8 Cambridgeshire and Peterborough NHS Foundation Trust, Elizabeth House, Fulbourn Hospital, Cambridge, United Kingdom; University of Cambridge, United Kingdom

## Abstract

Delusions are the persistent and often bizarre beliefs that characterise psychosis. Previous studies have suggested that their emergence may be explained by disturbances in prediction error-dependent learning. Here we set up complementary studies in order to examine whether such a disturbance also modulates memory reconsolidation and hence explains their remarkable persistence. First, we quantified individual brain responses to prediction error in a causal learning task in 18 human subjects (8 female). Next, a placebo-controlled within-subjects study of the impact of ketamine was set up on the same individuals. We determined the influence of this NMDA receptor antagonist (previously shown to induce aberrant prediction error signal and lead to transient alterations in perception and belief) on the evolution of a fear memory over a 72 hour period: they initially underwent Pavlovian fear conditioning; 24 hours later, during ketamine or placebo administration, the conditioned stimulus (CS) was presented once, without reinforcement; memory strength was then tested again 24 hours later. Re-presentation of the CS under ketamine led to a stronger subsequent memory than under placebo. Moreover, the degree of strengthening correlated with individual vulnerability to ketamine's psychotogenic effects and with prediction error brain signal. This finding was partially replicated in an independent sample with an appetitive learning procedure (in 8 human subjects, 4 female). These results suggest a link between altered prediction error, memory strength and psychosis. They point to a core disruption that may explain not only the emergence of delusional beliefs but also their persistence.

## Introduction

Associative learning forms the basis for belief formation [1]. It is driven by prediction error (PE) [1], [2], and there is evidence that altered PE signal drives the formation of delusions, the abnormal beliefs that characterise mental illnesses like schizophrenia [3]–[5]. PE represents the mismatch between what we expect in a given situation and what we experience [6]. It guides learning directly; we form and strengthen explanatory associations (e.g. between causes and effects) by minimizing PE [6]. In addition, PE guides the allocation of attention [7]; we attend to and learn about stimuli with unpredictable consequences. PE is represented in a range of neural structures and circuits, most notably the midbrain dopamine cells in area A10, the striatum and the prefrontal cortex [8]. In addition to guiding learning about rewards, PEs are vital to social [9] and perceptual learning [10] as well as the formation of causal beliefs [1], [2]. Based on these normative observations, we have proposed [3], [11]–[13] that, if PE signals occur inappropriately, individuals would attend to and learn about stimuli, thoughts and percepts that others would ignore. As a consequence, they would develop beliefs that do not reflect the contingencies of the real world – delusions. There is growing evidence that this may be the case [3]–[5]: people with delusions exhibit inappropriate prediction error signals whose magnitude correlates with delusion severity.

This model accounts for why delusions emerge but not for why they persist. We argue [13]–[15] that disturbed PE, as well as leading to erroneous updating of beliefs (and, hence, the emergence of delusional ideas) may also be critical in their persistence [13]–[15]. While this may appear counter-intuitive, given that PE is associated with the flexible updating of learned expectations [6], latterly it has been demonstrated that PE-driven memory reconsolidation can strengthen memories in the absence of reinforcement: specifically, the surprising re-presentation of a retrieval cue can strengthen a memory [16]. Reconsolidation occurs when memories are recalled into a labile state, integrated with new information, and consolidated once more [17], [18]. This process depends on PE [19], that is, surprising information returns related memories into a labile state [20]. Two competing processes are evoked [21]: on the one hand, there is extinction initiated by a negative PE signal, engendering competing learning [22] that overrides the original memory [23]. Extinction memories involve new learning, not simply forgetting of the old representation (as demonstrated by the fact that memories of the reinforced situation can recur spontaneously in rats and humans [23], [24]). Negative PE signals guide this new learning [22]. On the other hand, the positive PE response to predictive cues [8], [25], [26] engages an expectation of reinforcement and leads to the reminder-based strengthening of the belief [21]. The precise balance between these processes is driven by the magnitude, sign and timing of the accompanying PE signal [19].

Given the growing evidence that the psychotic state is associated with aberrant PE [3], [4], [27]–[31], we have suggested that this extinction-reconsolidation balance may be fundamentally altered in delusions [14]. That is, altered PE signal leads patients to attend to and learn about events that healthy individuals would ignore [3], [11]–[14], [31], [32]. This accounts for the emergence of delusions, which form as explanatory schemes [33]. But the same disturbance means that the delusion will be frequently reactivated, employed to explain subsequent experiences [34], and strengthened once more [13], [14], [35]. This strengthening may share a cognitive basis with the *illusory truth effect*, whereby judging the veracity of a statement enhances later belief in its truth [36]. Patients with delusions are more susceptible to the illusory truth effect, particularly for judgments related to their delusions [37]. We hypothesise that in psychosis, aberrant PE signal encourages inappropriate memory strengthening and delusion fixity, relative to extinction [14].

In short, we suggest that the same disturbance in PE may account for both the emergence and the persistence of delusions.

**Figure 1:**
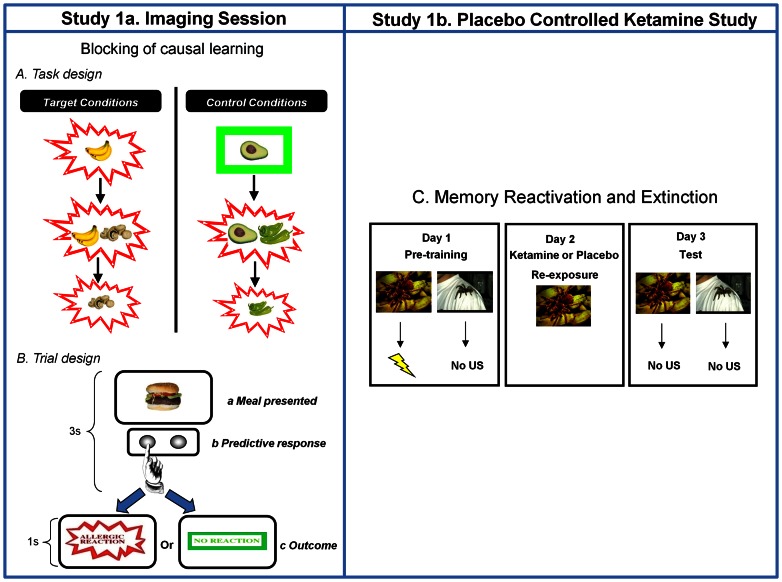
Study Design. *A. Task Design.* Target and control conditions for the food-allergy causal learning tasks. Subjects see that *bananas* cause an allergy in their patient. Subsequently they see that *bananas* and *mushrooms* cause the allergy. Their prior learning about bananas should block new learning about the mushrooms. In the final phase of training, subjects see the mushrooms causing the allergy; this violates any blocking that took place in the previous Stage. Blocking trials are compared to control events that are matched for the presence of allergy as well as novelty and familiarity (*Avocado and Chilies*). Likewise, at Stage 3, there are trials matched for novelty and familiarity that act as comparators for the blocking violation events. The figure depicts key trial types. Filler cues were also presented to control for the relative frequency of allergic reactions. Each trial type was presented 10 times for Stage 1 and 6 times for Stages 2 and 3 with the caveat that no trial type was repeated before all trial types had been presented. *B. Trial Design.* On each trial, subjects saw a meal that their patient had eaten for 3 seconds. During this time, they made a prediction response – pushing one button to predict an allergy and another to predict no allergy. They also held the button down for longer the more confident they were that they were making the right choice. Next they were shown the effect of that meal on their patient. If he suffered an allergy, they would see the words *Allergic Reaction* in red letters with a jagged border for 1 second. If there was no allergy, subjects saw the words *No Allergy* in green letters with a green rectangle around it for one second. *C. Memory Reactivation and Extinction Task.* In a follow up placebo-controlled behavioral study of ketamine, the same subjects from the scanning study attended the laboratory on three consecutive days twice (separated by at least one month). On Day 1, they learned that one visual stimulus predicted the delivery of a 90dB loud noise with 70% contingency and another cue never predicted load noise. The following day (Day 2) they saw the noise predicting cue whilst receiving an infusion of either ketamine or placebo. The next day (Day 3) they returned to the lab and observed the cues again in extinction. After 30 trials of extinction, they were reminded of the loud noise once more and observed a further five repetitions of each cue in extinction. We tracked skin conductance responses to the cues on Day 1 and Day 3. The subjects returned to repeat the procedure with different cues and received the other infusion the second time around.

We tested this hypothesis using a drug model of psychosis [38], [39]. In a previously published fMRI study, we quantified individual neural responses to PE [40]. The same participants were recruited to a placebo-controlled ketamine study, in which we assayed the psychotomimetic effects of ketamine as well as its impact upon the re-presentation of an aversive CS conditioned 24 hours previously (see Figure 1). We initially chose aversive learning and memory reconsolidation because, of the few reconsolidation studies in human subjects [20], [41]–[44], none have previously examined appetitive memory. We hypothesized that, since ketamine engenders aberrant PE signal in response to unsurprising events [38], it should induce reactivation and strengthening of memories, and this effect should be directly relatable to individual variability in PE signal, measured using fMRI, as well as to the severity of ketamine-induced symptoms. Given that it could be argued that any effect of ketamine could be attributed to generalization of the unpleasant psychotomimetic experience to the reactivated cue, we conducted a follow-up behavioral study of appetitive conditioning (Study 2) in which an independent sample of subjects.

We carried out two studies: Study 1, in which we relate PE brain responses, Ketamine-induced psychopathology and ketamine effects on aversive memory reconsolidation, and Study 2, in which we examine ketamine effects on reactivated appetitive memories in a separate cohort of subjects. To be clear, the fMRI study (Study 1a) was not set up to characterize the neural correlates of memory reactivation or reconsolidation [44], [45]. Rather it was conducted to capture individual differences in PE brain response. Those individual differences then formed the basis for exploring how variability in PE relates to the effects of ketamine on memory reconsolidation and the genesis of psychosis-like symptoms (Study 1b). A relationship between the neural marker for PE and reconsolidation across our studies would constitute evidence in favor of our hypothesis: PE disturbance is key to the formation and maintenance of delusions [14]. Study 2 aimed to clarify the interpretation of Study 1b.

## Methods

The study was approved by the Cambridge Local Research and Ethics Committee and was carried out in accordance with The Code of Ethics of the World Medical Association (Declaration of Helsinki). All subjects gave written informed consent.

### Study 1 – Subjects

18 (8 female) right-handed, healthy volunteers (aged 19–28) were recruited through local advertisement. No subjects reported a history of psychiatric illness, drug abuse or contra-indications for MRI. We excluded subjects with any history of alcoholism as well as current smokers [46]. One subject was excluded upon discovery of a past history of psychiatric illness. Participants completed other cognitive tasks under ketamine (examining spatial learning and memory, bodily agency, instrumental agency and visual perception) [47], [48]. Further observations will be reported elsewhere. For Stage 1 (fMRI), participants attended for testing on one occasion. For Stage 2, they attended on a total of six occasions (see below).

Study 1a comprised the fMRI study and 1b the placebo-controlled ketamine challenge. For each participant these were separated by a minimum of 4 weeks.

#### Study 1a – Functional Neuroimaging of PE signal

For Study 1, the fMRI stage (Study 1a) involved a Kamin blocking task [49] where initial causal learning blocked subsequent causal associations from forming to a contingent but redundant stimulus (see Figure 1). Blocking is a cornerstone of PE driven learning theories [8]; in the absence of PE, there is no learning about the blocked cue [50]. However, individuals with psychosis show attenuated blocking [51] as do healthy subjects treated with amphetamine [52], consistent with an aberrant PE account of psychosis [3], [13], [32], [38]. Individuals showing weaker blocking have “inappropriate” PE, driving them to attend to and learn about the redundant blocked cue. This learning was captured behaviorally (the degree to which subjects made confident predictions about the blocked cue) and neurally (the magnitude of brain surprise response to trials that violate blocking). That is, subjects with stronger blocking should be more surprised to see the blocked cue predicting a salient outcome, while those with weaker blocking should be less surprised. The present procedure has some similarities with the unblocking effect, previously demonstrated in rodent conditioning [53] and human causal learning [54] and used recently in experimental animals to clarify the neural mechanisms of reward prediction and PE [55]. Although our task does not involve reward per se, it has previously been used to emphasize the importance of neural and behavioral PE mechanisms in the formation of causal beliefs about the world in the absence of explicit reward [1], [2]. As in previous work, neural PE responses to events that violated what had previously been learned were used as a marker for the strength of previous learning [2]. Furthermore, we assayed brain responses during blocking trials (compared with control events matched for novelty, familiarity and contingency with the outcome) as a further metric of aberrant PE – those subjects who imbue the redundant blocked cue with causal significance ought to have responses in PE-related regions during blocking trials – providing a neural metric of their aberrant PE at the time of the redundant trials.

We used an established causal learning approach, in which learned expectations are violated to produce a prediction error [2]. We examined Kamin blocking, in which prior learning interferes with what is subsequently acquired [49]. Subjects were asked to imagine themselves working as an allergist confronted with a new patient “*Mr. X*”. Trials comprised presentation of a food picture (representing a meal eaten by Mr. X), a predictive button push response by the subject and, following this, an allergic-reaction or no reaction outcome. Subjects held the button down longer the more confident they felt in their prediction [2], providing a sensitive assay of learning as follows:
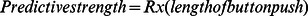
R is the predictive response (coded by +1 for prediction of an allergy and –1 for prediction of no allergy). The blocked cue induces a near zero score, since subjects should not learn about it.

#### Trial sequence

Training consisted of three phases: *Learning*; in which prior expectancies were developed, *Blocking*; in which those prior expectancies ‘blocked’ new learning, and *Violation*; which provided a metric for the strength of blocking. In brief, the key trials in Stage 1, *Learning*, comprised of single foods that caused or did not cause the allergy. There were also meals presented in this stage, which comprised of two different foods and were employed as fillers such that subjects were not surprised by the appearance of meals of two foods during the subsequent blocking phase. In the *Blocking* phase, single items from Stage 1 were paired with novel food items. Having previously learned that a food from Stage 1 caused the allergy should block new learning to the novel food. Two separate blocking contingencies were presented, represented by different foods. In the blocking control condition, which served as a comparator in our imaging analysis (see below) a food that previously predicted no allergy was paired with a novel food and this compound caused the allergic reaction. In the final *Violation* phase, one blocked cue was shown causing the allergic reaction; responses to this cue were compared with those to the novel blocking control food leading to the allergic reaction (See Figure 1 for more information). In order to control for novelty, familiarity and contingency reversal, one of the cues subjected to blocking at Stage 2 was presented causing no allergy. Other filler cues were presented to balance for the presence and absence of an allergic reaction, across trials involving single foods and compounds of two foods. The food stimuli used were identical to those used in our prior work [2], [3], [38], [56]. As in previous studies, the role played by the cues was randomized and counterbalanced across subjects such that variance in salience of the cues and their contingency with the outcomes were not confounded [2], [3], [38], [56].

We focused our behavioral analyses on confirming that blocking did indeed occur in our subjects. To this end, we planned a paired t-test on subjects' prediction confidence for the first trial of Stage 3 on which they see the blocked cue alone (mushrooms in Figure 1) compared with their initial prediction the first time they saw the blocking control cue alone (chilies in Figure 1). Mean predictive confidence ratings were calculated such that subjects' responses to the initial presentations of blocking and control cues for both contingencies (confirmed and violated) both contributed to the behavioral analysis. This was legitimate because up until this point (i.e., before subjects saw the outcome at the first trial of Stage 3), the novelty, familiarity and contingency with the outcome of these parallel causal contingencies were identical.

### fMRI Data Acquisition

We used a Siemens Trio scanner operating at 3 Tesla. 720 gradient echo T2*-weighted echo-planar images depicting blood oxygenation level-dependent contrast were acquired for each subject. The first seven images were discarded to avoid T1 equilibration effects. The remaining images covered the three task phases that ran continuously, in series; Stage 1 (Learning, 10 repetitions of each trial type) followed by Stage 2 (Blocking, 6 repetitions of each trial type) followed by Stage 3 (Violation, 6 repetitions of each trial type). Images were positioned parallel to the anterior commissure–posterior commissural line and comprised 35 slices, each of 2 mm with a 0.5 mm interslice gap. A repetition time of 1620 ms was used with an echo time of 30 ms and 90° flip angle. The scanner had a 192 mm field of view with a 64×64 data matrix.

### fMRI Data Analysis

fMRI data were analyzed using SPM5 (Wellcome Department of Cognitive Neurology, London, UK; http://www.fil.ion.ucl.ac.uk/spm). Images were realigned, spatially normalized to a standard template and spatially smoothed with a Gaussian kernel (8 mm at full width half maximum). The time series in each session were high-pass filtered (to a maximum of 1/120 Hz) and serial autocorrelations were estimated using an AR(1) model. The average haemodynamic response to each event was designated at the presentation of the outcome. Trials were modeled using a canonical, synthetic haemodynamic response function used as a covariate in a general linear model. A parameter estimate was generated for each voxel for each event. Responses were parametrically modulated by the subjects' confidence in their prediction for that event. Individuals' contrast images, derived from the pair-wise comparisons between key events, were then entered into a second-level group analysis for each of the stages. Given our a priori hypotheses and prior work [3], [38], we used the PickAtlas tool [57] to confine analyses to a single mask comprised of a series of regions of interest (ROI), total volume 1805 voxels: The five ROIs that comprised our mask were: right lateral prefrontal cortex (rPFC, a sphere of radius 10 mm centered on 50, 30, 28, the centroid generated by averaging across our prior studies of causal reasoning [2], [38], [56], [58]), left and right striatum and left and right substantia nigra (defined anatomically using the tool [57]). Hence, responses and relationships in these regions were tested simultaneously by applying the mask.

Brain responses to events that violated blocking (i.e. events when the blocked cue was shown causing the allergy, *Mushrooms* in Figure 1) were compared with unsurprising control cues (*Chili* in figure 1). Subjects who blocked most should be most surprised by the blocked cue causing the allergy, indexed as more extensive frontostriatal activation in response to such trials.

We also identified brain responses to blocking trials (*banana* and *mushrooms,* Figure 1) relative to matched control events (*avocado* and *chilies*, Figure 1). This comparison revealed the brain regions engaged whilst blocking was taking place.

We aimed to determine the relevance of individual PE-responsiveness to the effects of ketamine on subsequent memory expression. Therefore, we computed correlations between phase 3 violation-related activation in the key ROIs (all 5 ROIs were combined into a single mask, volume 1805 voxels) and the effects of ketamine on subjective cognitive task performance applying small volume correction for multiple comparisons [59]. For each correlation we report the z-score in the particular regions implicated. All reported findings were associated with false discovery rate corrected p-values less than 0.05 [60] across the entire mask which comprised all ROIs. For illustrative purposes we plot the relationships between brain responses and behavioral ratings. We are aware of the potential for statistical non-independence or circularity in correlative analysis [61] and hence we do not re-compute Pearson's r-values for the relationship between the parameter estimates from our fMRI models and the cognitive measures of interest.

We computed the difference between the magnitude of ratings and Galvanic Skin Responses (GSR) to the cue reactivated under ketamine and its counterpart reactivated under placebo for each subject. Post-reactivation GSR responses were defined as the average response to the first block of 5 trials in extinction on Day 3. Ratings of valence and arousal were taken at the end of the extinction session and corrected by the ratings at the end of Day 1.

### Study 1b – the effect of ketamine on memory reactivation

In the drug study (Study 1b, separated from Study 1a by at least 4 weeks), we employed ketamine as means of engendering psychotogenic aberrant PEs and we assayed the effects of reactivating a memory in the presence of such signals. As well as reporting their psychosis-like experiences (captured using a standard rating scale – see below), subjects completed a reconsolidation task involving initial Pavlovian conditioning of a picture stimulus as a predictor of an aversive auditory stimulus followed by re-presentation of the picture cue 24 hours later during ketamine or placebo infusion. The day after ketamine or placebo infusion, we tracked subjects' responses to the picture cue in extinction (i.e. in the absence of the aversive auditory stimulus).

The study was a double-blind, placebo controlled, randomized, within-subjects investigation of the effects of intravenous ketamine (the order of placebo and ketamine infusions was counterbalanced across subjects). An un-blinded clinician administered the infusions, however those administering cognitive tests and acquiring symptoms ratings remained blind. Subjects attended on two main study visits, once for drug, the other for placebo infusion. On the drug day, subjects received a computerized target controlled infusion of ketamine (200 ng/ml plasma) whilst they performed a series of cognitive tasks and a clinical interview exploring the presence, nature and severity of any psychotic symptoms. On the placebo day, a saline infusion was administered whilst subjects performed parallel versions of the cognitive tests and clinical interviews. There was a one-month washout period between drug and placebo visits to avoid effects related to activity of ketamine's metabolites.

#### Infusion protocol

Intra-venous catheters were inserted into the forearms, bilaterally, for ketamine infusion and serial blood sampling. Racemic ketamine (2 mg/ml) was administered by target-controlled infusion system using of a Graseby 3500 syringe driver pump (Graseby Medical Ltd, UK) under the control of Stanpump software (Freely available courtesy of Shafer S. http://opentci.org/doku.php?id=code:code). Steady state concentrations were implemented by administering a bolus, followed by an infusion whose rate was recalculated every 10 seconds; designed to replace drug removed by redistribution and metabolism. The infusion rates required for the bolus and maintenance infusion rates were determined by an algorithm in Stanpump, using a pharmacokinetic model for ketamine.

#### Fear conditioning, Reactivation and Extinction

This aspect of the procedure required six visits (three for placebo, three for ketamine). The day before the ketamine or placebo session (Figure 1, Day 1), subjects underwent aversive conditioning; learning about two visual conditioned stimuli (CS_1_ and CS_2_, each presented on 30 occasions). These were pictures of spiders, to which fear conditioning accrues readily [41], [62], [63].

On each trial, a cue appeared on screen for 4000 msec. CS_1_ predicted the delivery of a 750 msec 90dB noise unconditioned stimulus (US) through noise canceling headphones on 70% of trials. CS_2_ never predicted the US. We measured skin conductance responses from the non-dominant left hand. There was a mean inter-trial interval of 10 seconds. At the end of conditioning on experimental Day 1, subjects rated the two cues for valence and arousal.

On the drug or placebo infusion day (Figure 1, Day 2), subjects were fitted with the electrodes for recording skin conductance responses and the headphones for delivery of aversive USs. They were shown one single instance of CS_1_. The aversive noise was not presented. Their skin conductance was not recorded during this single trial.

The day after the ketamine or placebo session (Figure 1, Day 3), CS_1_ and CS_2_ were presented a further 20 times, and neither cue predicted the US. Subsequently five un-cued USs were played and the cues were each presented again five times to quantify reinstatement of responding [41].

Since memory reconsolidation and extinction appear to be competing processes [14], [21] and differentially sensitive to reminders of the unconditioned stimulus [41], [42], we re-presented the aversive auditory stimulus followed by further extinction training through which we quantified the degree to which responding to the predictive cue could be re-evoked and re-extinguished, reasoning that reminders of the unconditioned stimulus should re-evoke responding to the conditioned stimulus [41]. If a memory cannot be reinstated, then we can be more confident that its reconsolidation has been blocked [41]. On the other hand, if responding is reinstated more strongly for the ketamine-reactivated cue than the placebo-reactivated cue, we can be more confident that we enhanced memory strength via reconsolidation [64].

At the end of the procedure, subjects rated the cues for valence and arousal.

Subjects' GSR responses were recorded using 9 mm Ag/AgCl electrodes filled with electrolyte paste and placed on the hypothenar surface and a BIOPAC MP150 system acquiring at a frequency of 50 Hz in concert with a PC running Acknowledge software (version 3.7).

We designated the 2 seconds prior to the onset of an event as a baseline and recorded the mean galvanic skin conductance response in microsiemen (µS). For each event, we identified an 8 second period following its onset and recorded the maximum skin conductance in µS [65]. We subtracted the baseline from the maximum response for each event thus identifying the skin conductance response to each event. To confirm acquisition of differential conditioning, we computed the mean GSR response to the final 5 CS_1_ trials and the final 5 CS_2_ trials on Day 1. For analysis of the 20-trial extinction session, we calculated a mean skin conductance score for 4 blocks of 5 trials comprising the 20 extinction trials. To examine reinstatement, we computed GSR responses to each of the final 5 non-reinforced CS_1_ and CS_2_ trials.

#### Clinical Interview

Subjects' symptoms were rated using the clinician administered dissociative states scale (CADSS) [66].

### Planned analyses: Linking study 1a with study 1b

We sought to examine the relationship between the neural, behavioral and clinical datasets to test our hypothesis – that variation in prediction error signal would correlate with the effects of ketamine on memory reconsolidation and that subjects' behavioral responses following the reconsolidation manipulation would relate to the severity of their psychosis-like symptoms. Given that multiple experiments were conducted to examine this hypothesis, the potential for type-I error is high. We avoided this potential by focusing our correlational analyses on our *a-priori* predictions. First, our prior work related PE signal with the perceptual aberration sub-scale of the CADSS [38], hence, when relating imaging or behavioral findings to clinical data, we only examined this subscale. Next, our primary dependent variables coding the impact of ketamine during reactivation on reconsolidation were three-fold – subjects' GSR responses during initial extinction and their ratings of valence and arousal post-extinction. In order to limit our exposure to type-I error, we planned only to bring through the variables (of those 3) that were significantly impacted by ketamine to the regression analysis. We did not relate the reinstatement data to the neural or clinical data as they were gathered in order to confirm and inform upon any behavioral effects that we observed during extinction (i.e. whether or not we had modulated reconsolidation or blocked extinction). Finally, our plan was hierarchical; we related the neural and behavioral data (again, only those variables that were significantly impacted by ketamine), given any significant associations, we then explored the link between those behavioral variables and the clinical data (summarized by the perceptual subscale).

Therefore, the maximum number of potential regression analyses we could have computed was 6: three separate regressions of neural data on GSR, Valence and Arousal and three regressions of CADSS perceptual subscale on GSR, Valence and Arousal. In practice, given the results we obtained (see below), we computed four correlations: 2 between brain and behavioral data, and 2 between behavioral and clinical scales. These analyses harnessed the power of individual differences across subjects, constrained however, by our prior work [3], [38] and hypotheses [14].

### Study 2 – Ketamine's effects on reconsolidation of appetitive memory

As in Study 1b, this study employed a double-blind, placebo controlled, randomized, within-subjects design to assess the effects of intravenous ketamine on memory reactivation and subsequent reconsolidation, appetitive memory for juice rewards in this case. Subjects were 8 healthy volunteers who met the inclusion exclusion criteria for experiment 1 (4 female, aged 19–33).

As before, subjects attended on two main study sessions, once for ketamine, the other for placebo infusion. On the drug day, subjects received an infusion of ketamine (200 ng/ml plasma, administered by an un-blinded clinician exactly as in experiment 1) whilst they performed a series of cognitive tasks (to be reported elsewhere) and a clinical interview exploring the presence, nature and severity of any psychotic symptoms. On the placebo day, a saline infusion was administered whilst subjects performed parallel versions of the cognitive tests and clinical interviews. There was a one-month washout period between drug and placebo visits to avoid effects related to activity of ketamine's metabolites.

#### Reconsolidation of appetitive memories

The reconsolidation memory design followed the structure of experiment 1 closely. Subjects attended the laboratory on 6 occasions, 3 for the drug arm of the study and 3 for the placebo arm. Drug and placebo were administered in a randomized counterbalanced order.

On Day 1 the participant sat in front of a computer screen, holding four reward delivery tubes in their mouth. Three of these tubes, before reaching the mouth, converged on a 3 to 1 valve, allowing three different rewarding liquids to be received through one emerging tube that formed a mouthpiece and ensured the participant was comfortable during the task.

One of three visual stimuli (conditioned stimulus – CS) was presented on a computer screen, followed by the delivery of a liquid reward (unconditioned stimulus – US) corresponding to that CS. One of three NE-510 OEM High Pressure Syringe Pumps delivered 0.9 ml of liquid reward per infusion, through each delivery tube. Visual CS presentation and liquid US delivery were controlled using in-house computer software.

One cue predicted the delivery of blackcurrant juice drink, another the delivery of orange juice drink, and the third predicted water delivery. The first two cue-outcome pairings engendered positive associations between the previously neutral cues. The water US served as a neutral outcome. Each cue-outcome pairing was presented 25 times.

The final tube attached to the mouthpiece allowed monitoring of the pressure changes that occurred throughout the trial (to this end, participants are instructed to use their lips to form a tight seal round both tubes throughout the task). Pressure measurements were made with a Biopac™ data acquisition system, which amplified and digitized the pressure signal that was then recorded using the ‘Acknowledge™ 3.9.0’ software package.

During CS presentation, subjects anticipated how much they would like liquid they were about to receive by selecting a number between 1 and 9 on a computer keyboard, during presentation of the CS (1 =  dislike, 9 = like very much).

On Day 2, whilst looking at the computer screen, the participant held the tubes in their mouth as per Day 1. However, just one trial occurs. The CS that predicted juice reward (either blackcurrant or orange randomized and counterbalanced across subjects) was presented and subjects made their anticipatory liking rating. Pressure changes were not measured.

On Day 3, participants held the tubes in their mouths and were presented with the CSs as per the learning phase. They were instructed to rate their anticipated liking of the liquid rewards, from 1 – 9, as they had in the learning phase. However, no liquids are delivered. Each CS was presented 15 times. Their anticipatory sucking during the CSs was also recorded.

## Results

### Study 1

#### Study 1a: Behavioral results

**Figure 2:**
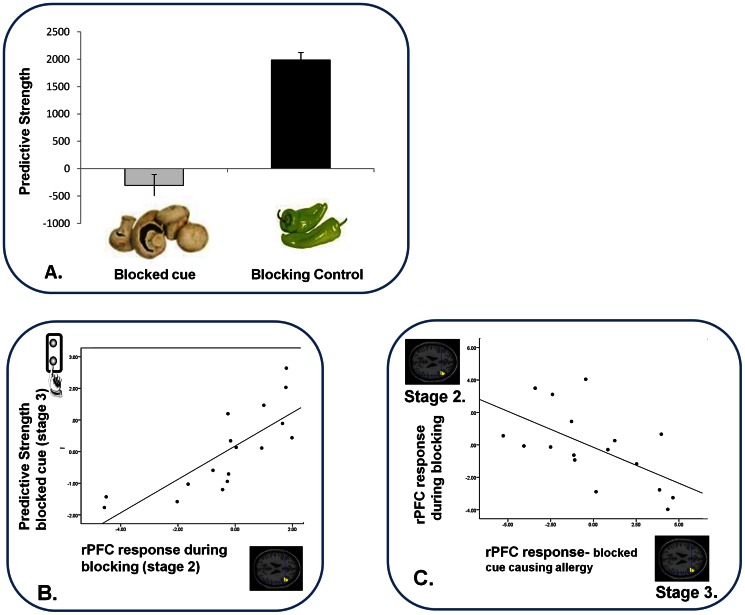
Blocking Behavior and its Relation to Brain Responses. *A. Behavioral Predictions For Blocked and Control Cues.* Subjects predicted with low confidence about the blocked cue, when exposed to it at Stage 3; confirming that blocking had taken place. Error bars represent SEM. Y-axis represents subjects' predictive strength; their degree of confidence (duration of predictive button push response) multiplied by correctness of their prediction. Hence lower scores reflect uncertain and unstable predictions, which we observed to blocked cues when compared with blocking control cues (whose causal association with the allergy is more robust). *B. Relating Predictions about the Blocked Cue (Stage 3) to Blocking Responses.* Subjects who showed the lowest confidence when predicting what would happen following the blocked cue had the most attenuated right DLPFC response during blocking trials. X-axis represents the right DLPFC parameter estimates extracted from a contrast image comparing blocking trials with blocking control trials. Y-axis represents subjects' behavioral predictions about the blocked cues prior to seeing their predictive outcomes at the first trials of Stage 3. *C. Relating Brain Responses During Blocking to those during Violation.* Subjects with the most attenuated DLPFC response during blocking showed the greatest right DLPFC response when that blocking contingency was subsequently violated. X-axis represents the right DLPFC response to observing the blocked cue causing the allergic response during Violation (Stage 3), compared with control event. Y-axis represents right DLPFC response to blocking trials compared with blocking-control trials.

Subjects showed clear evidence of behavioral blocking. They were less likely to predict an allergy when confronted with the blocked cue, and their predictions were less confident (t = 7.169, 2-tailed, d.f.  = 16, p<0.0001 (Figure 2A).

#### Study 1a: fMRI results

**Figure 3:**
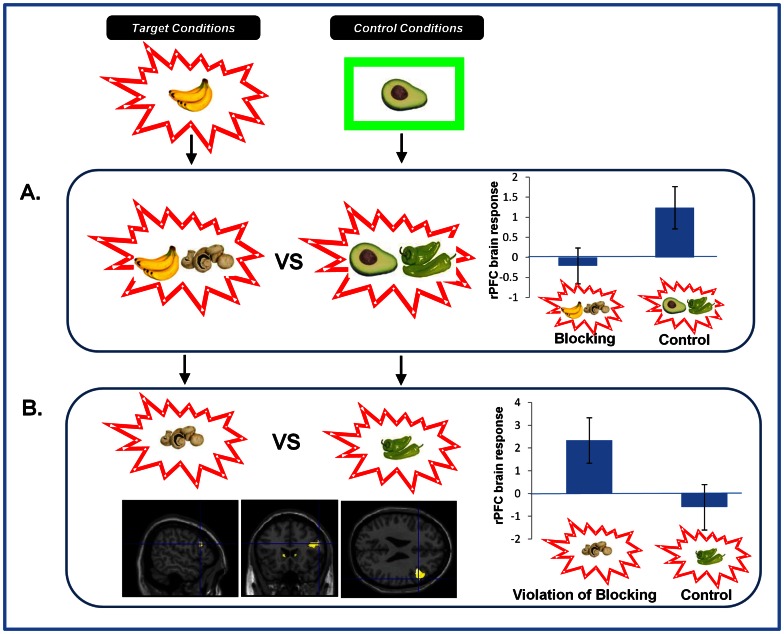
Brain Responses to Blocking and Violation of Blocking. *A. Brain responses to Blocking vs. Control Trials.* Right DLPFC responses to blocking trials were significantly attenuated compared with control trials. Parameter estimates extracted from SPM in arbitrary units. Error Bars represent standard error of the mean (SEM). *B. Brain Responses to the Violation of Blocking.* Violating the blocking at Stage 3 significantly engaged right DLPFC. Parameter estimates extracted from SPM in arbitrary units. Error bars represent SEM.

Presenting the blocked cue causing the allergy engendered a PE response in rPFC (x = 42, y = 18, z = 20. z-score  = 2.50. p<0.05) and bilateral head of caudate (x = −6, y = 16, z = 6. z-score  = 2.99, p<0.05; x = 4, y = 14, z = 6. z-score  = 2.23 p<0.05), when compared with control trials (Figure 3B). Blocking trials were associated with an attenuated response in rPFC relative to control trials (x = 42, y = 18, z = 20. p<0.05, Figure 3A). Subjects with the greatest reduction in rPFC response to blocking (Stage 2) showed the greatest subsequent evidence of blocking. That is, they showed a reduced tendency to predict an allergy in response to the blocked cue at Stage 3 (z = 3.96, p<0.05, Figure 2B). As well as the rPFC response to blocking predicting subsequent behavior, it also predicted the magnitude of brain response to the subsequent violation of blocking (z = 3.77, p<0.05 Figure 2C).

In brief, the behavioral and neural responses to blocking indicated the success of this experimental manipulation and were strongly consistent with our previous studies indicating a role for rPFC in error-dependent learning of causal associations. Having established this, the key question was whether variation across individuals in this PE response related to the effects of ketamine. From Study 1a we can extract a measure of variation PE driven belief formation between individuals. Study 1b had two aims: to assess the influence of ketamine administration upon processes related to belief persistence and to confirm that variation in ketamine effects was related to variation in the belief formation measure established in Study 1a.

### Study 1b – within-subject placebo-controlled study of ketamine

#### Study 1b: Ketamine plasma levels

The mean ketamine plasma concentration across subjects was 259.8 +/−85.6 ng/ml.

#### Study 1b: Psychopathology

Compared to placebo, ketamine increased perceptual aberrations (CADSS perceptual subscale score, t =  4.748, d.f.  = 14, p<0.0001), which are associated with delusion-like ideation on ketamine [38], [67], and phenomenologically similar to the delusional mood that portends delusion formation [33], [68]–[71].

#### Study 1b: Cognitive effects of ketamine

**Figure 4:**
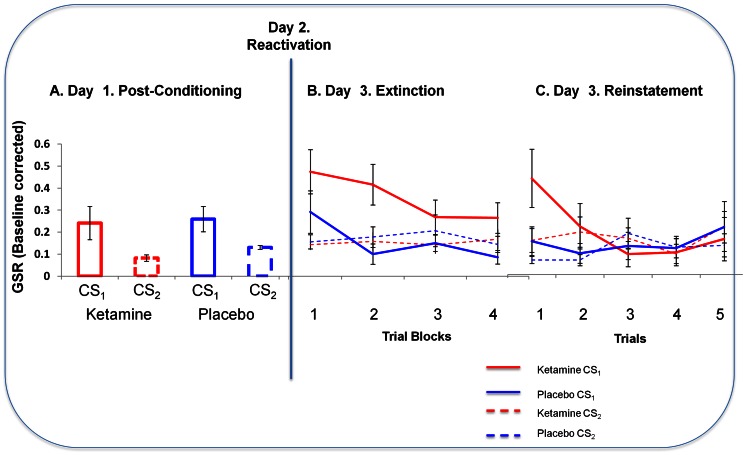
GSR Responses to Fear Cues Across Study Days. *A. Skin Conductance Responses to Cues after Initial Conditioning (Day 1).* Subjects means skin conductance responses to the final 3 trials at the end of initial conditioning on Day 1. Y-axis represents subjects Galvanic Skin Responses to the cues (ketamine in red, placebo in blue; solid lines represent the to be reactivated cues (CS_1_) and dashed lines represent CS_2_ (non-predictive and non-reactivated). Error bars represent SEM. *B. Skin Conductance Responses to Cues in Extinction (Day 3).* Subjects showed an elevated skin conductance response to the ketamine-reactivated cue compared with the cue reactivated under placebo. Error bars represent SEM. Line graph: Y-axis represents subjects GSR responses to blocks of four extinction trials to cues reactivated under ketamine and placebo. Ketamine data are shown in red, placebo in blue; solid lines represent the reactivated cues (CS_1_) and dashed lines represent CS_2_ (non-predictive and non-reactivated). Error bars represent SEM. *C. Skin Conductance Responses to Cues Following US reminder (Day 3, post extinction).* When subjects were re-exposed to the loud noise outcome and then presented with the cues 5 more times in extinction, responses to the ketamine reactivated cue returned most strongly. Y-axis represents galvanic skin conductance response. Ketamine data are shown in red, placebo in blue; solid lines represent the reactivated cues (CS_1_) and dashed lines represent CS_2_ (non-predictive and non-reactivated). Error bars represent SEM.

Conditioning (Day 1) Subjects' skin conductance responses were consistent with differential conditioning to CS_1_ versus CS_2_ on the days prior to ketamine and placebo infusion. Focusing on mean responses to the last five trials in which each cue was presented, ANOVA revealed a main effect of cue (CS_1_ vs. CS_2_, F_(1,15)_  = 9.273, p<0.01) but no difference between the cues that were to be reactivated under ketamine or placebo (F_(1,15)_  = ,0.557 p>0.4) and the difference between CS_1_ and CS_2_ (i.e. learning) was equivalent for the cues that were to be reactivated under ketamine and those to be reactivated under placebo (F_(1,15)_  = 0.111, p>0.7, Figure 4). Hence, any subsequent effects of drug administration on reactivated cues cannot be attributed to differential learning about the to-be-reactivated cues before they were reactivated.

### Extinction and reinstatement of conditioned cues reactivated under ketamine or placebo

#### Post-Extinction Behavioral ratings

Note that all of these results relate to the third day of testing, i.e., 24 hours after the aversively trained cue had been re-presented under either ketamine or placebo. The difference reported between ketamine and placebo refers to changes in the impact of these cues arising from the manipulation 24 hours earlier.

#### Pleasantness

ANOVA revealed neither a significant main effect of ketamine, nor a significant drug by task interaction in terms of how unpleasant the subjects found the cues.

#### Arousal

**Figure 5:**
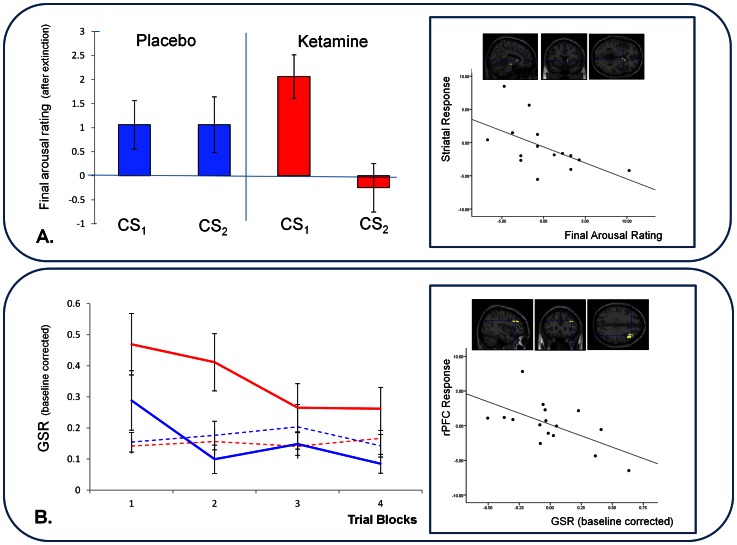
Responses to Fear Cues and their Relationship to PE Brain Responses. *A*. *Ratings of the Cues after Extinction and their relationship to Violation Responses*. Subjects rated the cue reactivated under ketamine as significantly more arousing than the cue reactivated under placebo. Those same subjects showed an aberrant striatal response during the violation of blocking indicative of inappropriate learning. Error bars represent SEM. Bar Graph: Y-axis represents subjects Final Arousal Ratings after the extinction trials, corrected by their initial ratings of the cues at baseline, such that residual ratings reflect conditioning. Scatterplot: X-axis represents those same Arousal ratings. Y-Axis represents parameter estimates extracted from right striatum from the contrast of blocking violation trials with their matched control events. *B. Skin Conductance Responses to Fear Cues and their relationship to violation Responses.* Subjects showed an elevated skin conductance response to the ketamine-reactivated cue compared with the cue reactivated under placebo. Subjects who showed the strongest skin conductance responses to cues reactivated under ketamine also showed the most inappropriate DLPFC response to the violation of blocking, indicating that they had learned inappropriately about the blocked cue. Error bars represent SEM. Line graph: Y-axis represents subjects GSR responses to blocks of four extinction trials to cues reactivated under ketamine and placebo. Scatterplot: X-axis represents the GSR to the first extinction trial (Ketamine minus placebo). Y-Axis represents parameter estimates extracted from right DLPFC from the contrast of blocking violation trials with their matched control events.

ANOVA revealed no significant main effect of ketamine on arousal ratings. However, there was a significant drug by task interaction; subjects rated the cue that had been associated with delivery of the loud noise presented to them under ketamine as more arousing than an equivalent cue that they had been presented under placebo (F_(1,15)_  = 4.69, p<0.05; Figure 5); reactivating an aversive memory under ketamine enhanced the salience of that memory.

### Post reactivation Psychophysiology: Extinction, Reminder and Reinstatement

We compared GSRs to the cue reactivated under ketamine with those to its counterpart reactivated under placebo using within subjects ANOVA. There was a significant main effect of task (F_(3,45)_  = 4.262, p<0.05); across blocks, responses extinguished. Crucially, there was a main effect of drug (F_(1,45)_  = 10.87, p<0.01): Skin conductance responses to the cue reactivated under ketamine were significantly higher than those to the cue reactivated under placebo (Figure 4B).

There was no significant effect of ketamine on responses to the loud noise US-reminders or subsequent habituation (data not shown). That is, having ketamine 24 hours previous did not alter subjects' responses to un-cued loud noises.

Within-subjects ANOVA revealed stronger reinstatement to the cue reactivated under ketamine manifest as a significant drug by task interaction, F_(1,15)_  = 2.113, p<0.05, Figure 4C).

#### Fear memory reactivation and prediction error

Subjects who rated the cue reactivated under ketamine as more arousing had a decreased PE response in the right striatum during the violation of blocking in the causal learning task, assayed 4-weeks earlier (z = 2.84, p<0.05, Figure 5A).

Subjects with larger skin conductance responses to the ketamine-reactivated cue demonstrated an attenuated prefrontal response to the violation of blocking during causal learning assayed one month previously (z = 2.86, p<0.05, Figure 5B).

#### Relating ketamine effects on memory reconsolidation to ketamine induced aberrant salience

**Figure 6:**
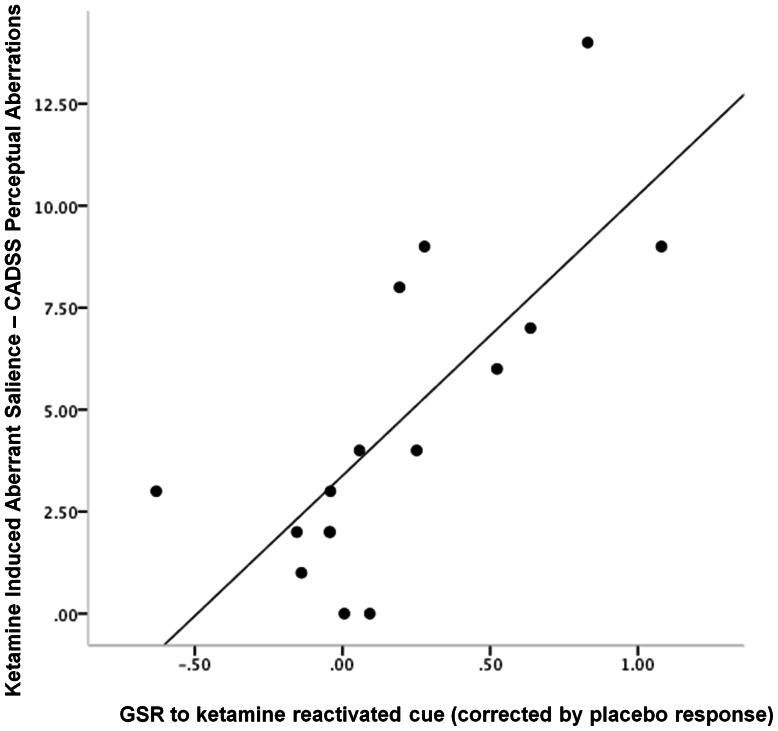
Relating Cue Responses to Psychotomimetic Response. *The relationship between ketamine induced aberrant salience and skin conductance responses to the cue reactivated under ketamine*. Subjects who experienced the most severe aberrant salience also showed the strongest skin conductance responses to the cue reactivated under ketamine. Plot features the difference in GSR response to the cue reactivated under ketamine from the cue reactivated under placebo on x-axis, CADSS perceptual subscale score (e.g., endorsing that background noises seemed louder, colors seem brighter, objects appeared to stick out from the background) on the y-axis.

Subjects who experienced most aberrant perceptual experiences (e.g., enhanced loudness of background noises) as measured by the perceptual subscale of the CADSS (related to aberrant PE in our prior work [38], and hence predicted to be involved *a priori*) showed the most ketamine-induced strengthening of their fear memories, manifest as an enhanced skin conductance to the re-presentation of the cue in extinction the day after the ketamine session (compared with placebo, r = 0.741, p<0.005, Figure 6).

### Post-hoc analysis: Relating Ketamine effects to brain responses during blocking

**Figure 7:**
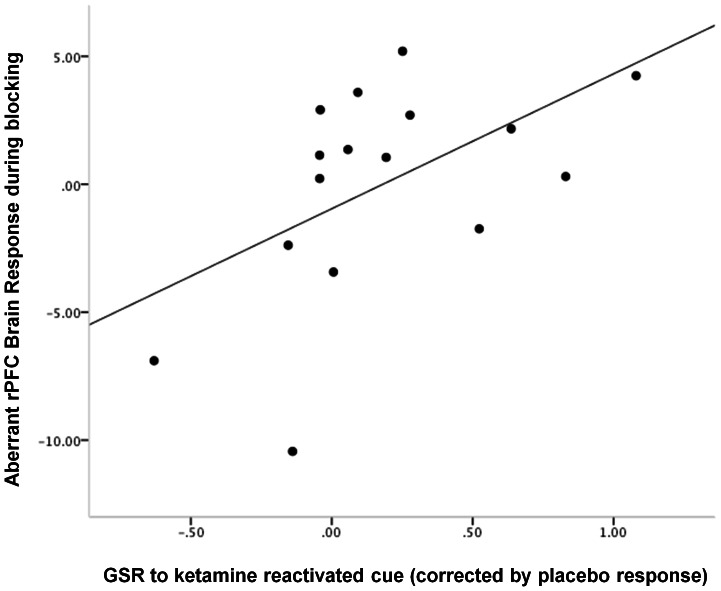
Relating Cue Responses to Aberrant PE During Blocking. The degree to which subjects inappropriately engage DLPFC during blocking trials correlated positively with their tendency to stronger GSR responses to ketamine-reactivated cues. This result is internally consistent with the Stage 3 finding – excessive responses during blocking and attenuated responses during its violation portend further memory strengthening in the context of ketamine. Plot features the difference in GSR response to the cue reactivated under ketamine from the cue reactivated under placebo on x-axis, rDLPFC responses during blocking trials (compared with control trials) on the y-axis.

Our measure of aberrant PE involved the brain response to the violation of blocking. As a test of internal consistency, we assayed the correlation between the memory strengthening effect of memory reactivation under ketamine and responses during the blocking trials. Consistent with the aberrant PE learning interpretation, rDLPFC response to blocking events correlated positively with enhanced GSR in extinction (z = 2.08, p<0.05, Figure 7).

The role of ketamine in enhancing persistence of memory for an aversive US in Study 1b might be due, in part or in full, to aversive aspects of the ketamine experience somehow enhancing the expression of CS-US knowledge, rather than the predicted influence on processes involved in extinction and reconsolidation.

Study 2 therefore was conducted to determine whether the effects of ketamine upon memory persistence would be observed for an appetitive US (consistent with a direct influence upon reconsolidation and extinction processes).

### Study 2 – Appetitive learning (behavioral study only)

This follow-up study clarified the effects of ketamine on post-reactivation memory processing.

Subjects' anticipatory ratings of pleasantness reflected that by the end of conditioning on Day 1, they had learned the CS-US relationships. ANOVA revealed a main effect of cue (F_(2,14)_ =  5.64, p<0.05), but no difference between the cues that were to be reactivated under ketamine compared to those that were to be reactivated under placebo 24 hours later (F_(1,7)_ = 0.151, p>0.7) and the difference between CS_1_ and CS_2_ was equivalent for the cues to be reactivated under ketamine and those to be reactivated under placebo (F_(2,14)_ =  0.88, p>0.4). In addition, the difference between anticipatory sucking pressure exerted to CS_1_ and CS_2_ was not significantly different across visits (paired sample t-test on difference in anticipatory sucking to CS_1_ and CS_2_, d.f.  = 7, t = 0.111, p>0.9), suggesting that this metric also indicated equivalent differential conditioning on Day 1 for ketamine and placebo.

Hence, subjects learned that the appetitive cues signified valued juices rather than neutral solution. Furthermore, they did so to an equivalent extent for cues associated with the ketamine session and those associated with the placebo visit. Any differences observed following reactivation could not be attributed to differences in prior conditioning.

**Figure 8:**
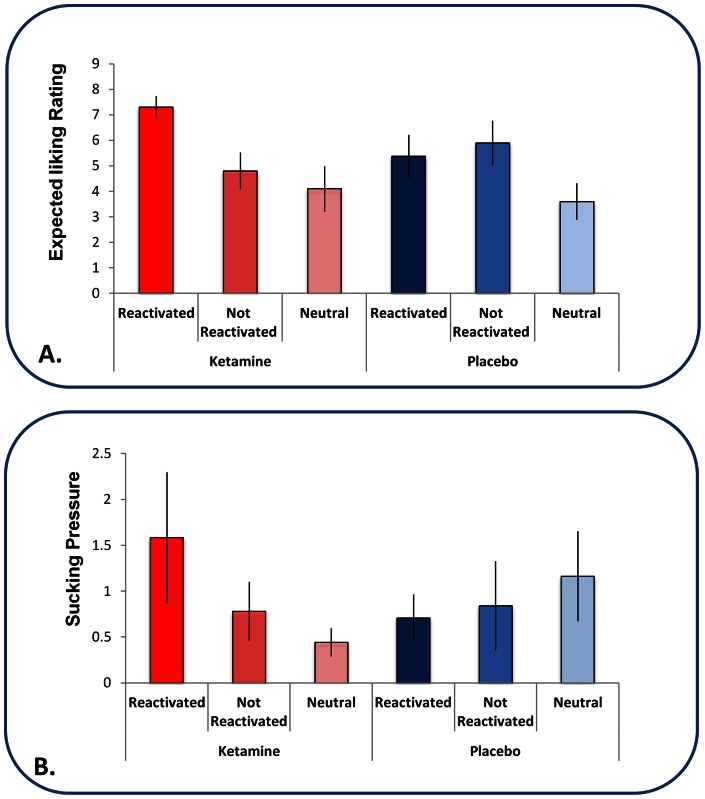
Appetitive Memory Reactivation. *A. Liking ratings of the liquid predicting cues*. Behavioral ratings of the cues on the first extinction trial (Day 3), following reactivation under ketamine or placebo (Day 2). Subjects liked the cue that had been reactivated under ketamine more than they liked the other cues and the cues from the placebo session. Error bars represent SEM. *B. Anticipatory sucking responses to the liquid predicting cues.* Anticipatory sucking in response to the first presentation of each cue type in extinction (Day 3) following reactivation under ketamine or placebo (Day 2). Subjects engaged in more anticipatory sucking prior to the cue that was reactivated under ketamine, the day after the ketamine session than the non-reactivated cues and the cues reactivated under placebo. Error bars represent SEM.

Repeated measures ANOVA of the pleasantness ratings subjects gave for the cues revealed a trend toward a significant drug by cue interaction (F_(2,14)_ = 2.715, p = 0.1). When we examined exactly the same contrast we conducted in our aversive memory experiment, comparing ketamine reactivation to placebo reactivation, there was a significant interaction (F_(1,7)_  = 6.377, p<0.05): cues reactivated under ketamine were rated as significantly more pleasant than those reactivated under placebo in extinction the following day (See Figure 8A). It is possible that association also retrieved the non-reactivated control cue during the re-activation on drug, hence the lack of significant interaction. However, the differences between ketamine and placebo reactivated positive cues are consistent with our findings from Study 1.

ANOVA revealed a significant drug by cue interaction (F_(2,14)_ = 4.074, p<0.05). Subjects applied greater sucking pressure during the first exposure to the cue reactivated under ketamine than its placebo reactivated counterpart as well as the un-reactivated salient and neutral cues (Figure 8B).

Given the relatively small sample size, we did not examine individual difference regression analyses for Study 2.

## Discussion

We observed that individual variability in the PE signal correlated with the strengthening of memories reactivated under ketamine and that this strengthening effect was correlated with the profundity of ketamine-induced perceptual disruptions. Furthermore, we partially replicated the strengthening effect in a new cohort of volunteers in whom appetitive memories were reactivated (there was only a trend towards drug by task interaction in the pleasantness ratings. The interaction was significant for the anticipatory sucking measures). Taken together, these findings support the hypothesized link between PE, retrieval-based memory modulation and psychopathology.

Our study demonstrated a clear blocking effect in human causal learning. During blocking trials, there was the expected [50] overall suppression of BOLD response in regions previously shown to be sensitive to PE [2], [3], [38], [56], [72]. When blocked cues were subsequently presented, group average predictive responses indicated that subjects had not learned to associate these cues with an outcome. Violation of that attenuated expectation was expressed neurally as a PE response in key frontostriatal regions [2]. Participants showing the lowest rPFC response during blocking trials showed the greatest subsequent behavioral evidence of blocking and the greatest subsequent neural “surprise” response when particular blocked cues proved unexpectedly to be predictors of an outcome (see Figure 2). More importantly, as we discuss below, variations in PE responding predicted individual vulnerability to the subsequent experiential and mnemonic impact of ketamine.

We determined the influence of ketamine on reactivated memories. Re-presentation of a pre-trained aversive cue under ketamine compared to placebo was associated with elevated ratings of unpleasantness and GSR when these were tested the following day. Crucially, individuals showing aberrant PE in the fMRI study were more susceptible to these enhanced effects. We argue that this underlines the importance of aberrant PE and subsequent memory processing in the pathophysiology of psychotic symptoms. Put simply, the failure of PFC to show an appropriate response to blocking (study 1a stage 2) and its ensuing violation (stage 3) are predictive of an enhanced vulnerability to the memory-altering effects of ketamine.

We considered the possibility that the strengthening of the reactivated cue occurred because the cue was associated with an aversive ketamine experience. However a similar (albeit weaker) effect in an appetitive conditioning in study 2 militates against this argument. For both aversive and appetitive reactivated cues, strength of responding (GSR and anticipatory sucking respectively) increased following ketamine. It is difficult to explain this observation in terms of attenuated extinction, since one would expect consistent responding from Day 1 to Day 3 for the ketamine reactivated cues and perhaps a decrease in responding for the placebo reactivated cue, which we did not observe.

It is perhaps surprising that ketamine (a non-competitive NMDA receptor antagonist with known amnestic effects [73]) appears to enhance reactivated memories. Initial pre-clinical studies of pharmacological effects on reconsolidation have demonstrated that NMDA receptor antagonism impairs or prevents memory reconsolidation. Importantly, however, only one rodent study employed ketamine as the NMDA blocking agent that perturbed drug-memory reconsolidation [74], others employ more potent NMDA receptor antagonists like MK-801 or PCP. The difference between our work and the rodent work may also have arisen because we are dealing with different response systems in human and rodent subjects [75], [76]. Behavioral studies capture numerous measures of conditioning, ranging from subjective ratings, skin conductance measures and startle responses to conscious expectancies of subsequent outcomes. In the intact organism, these measures often cross-validate. However, in a reactivation-degradation procedure in human subjects, only some of these measures are disrupted whilst other remain intact [42]: startle responses are degraded whereas measures related to conscious awareness are not. Electrodermal conditioning reflects anticipatory arousal [77]–[79] and conscious awareness of contingent relationships seems critical for the expression of skin conductance responses [77]–[79]. These measures were the focus of our present research and we did not record startle responses (perhaps more akin to freezing in pre-clinical models). Furthermore, in considering the fact that a ketamine-induced memory strengthening is counter-intuitive, it is notable that, ketamine, when used as an anesthetic in accident victims, may enhance PTSD symptoms [80], perhaps suggesting that aversive memories are enhanced, although this claim is controversial [81]. Our data suggest that both appetitive and aversive memories may be enhanced, depending on what is brought to mind during the ketamine experience. This must give us pause for thought when we consider the increasing interest in using repeated ketamine as an anti-depressant.

Intriguingly, in Study 1b, subjective and objective measures of the strength of fear conditioning correlated with variability in different components of the PE circuit: variation in electrodermal response to a fear-cue reactivated under ketamine was correlated with aberrant prefrontal PE responding whereas variation in the lingering subjective arousal ratings of the fear-cue reactivated under ketamine correlated with aberrant striatal responding. These results are perhaps indicative of different roles for PE across different regions: striatal signals may compute predictive value or salience, whereas PFC PE may sculpt more complex global expectations [82]. This distinction echoes that between model-free and model-based reinforcement learning [83], [84]. Both of these processes are driven by PE [82] and may be important for symptom generation and maintenance [3], [12]–[15]. These data provide preliminary support for our model; aberrant PE drives delusion formation by imbuing stimuli, thoughts and percepts with a salience that demands explanation. Such an explanation requires rumination and memory reconsolidation, strengthening it inappropriately such that it eventually becomes impervious to contradictory evidence [14].

Another important point to take into account is that, as well as blocking NMDA receptors, ketamine engenders glutamate release [85], which may stimulate AMPA receptors [85] and engage intracellular signaling cascades that engender synaptogenesis [86]. For example, ketamine increases prefrontal extracellular signal regulated kinase (ERK) [87], a key regulator of the fate of reactivated memories [88]. Furthermore, memantine, an NMDA antagonist like ketamine, has been shown to facilitate aversive memory reconsolidation even when given two hours prior to memory reactivation in day-old chicks [89].

Prior work has shown that ketamine increases cortical excitability [90], increasing AMPA receptor stimulation [91]. Recently, transcranial direct current stimulation, which also increases cortical excitability, has been shown to enhance the strength of reactivated memories [92]. Rodent work has confirmed the crucial role of AMPA receptor mobilization in this post-reactivation strengthening effect [93]. Our own computational modeling work suggests that ketamine induces dis-inhibition of cortical microcircuits resulting in synaptic glutamate spillover [94], [95]. In brain slices, this ketamine induced increase in glutamate release and EPSCs can be curtailed by propranolol [96], the beta-adrenergic receptor antagonist that has been shown to degrade reactivated memories across species [41]. Furthermore, the aberrant salience experiences and delusion-like ideas (that we presently relate to excessive PE driven memory reconsolidation) are ameliorated by lamotrigine, a drug that blocks presynaptic glutamate release and cortical excitability [97], [98]. Taken together, these data suggest that ketamine-induced psychosis results from excessive glutamate release which engages aberrant prediction errors and hence excessive memory strengthening (even if those memories are false).

**Figure 9:**
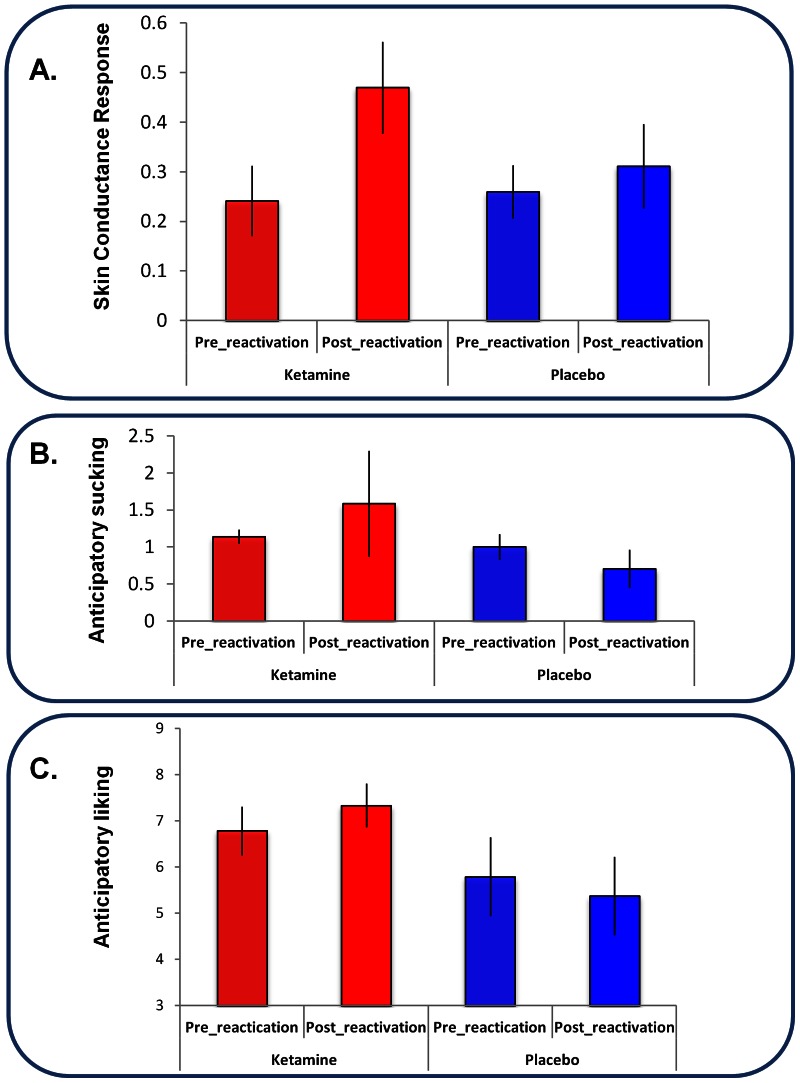
Memory Strengthening or Extinction Failure? *A. Skin Conductance Responses.* Skin conductance responses to the CS+ at the end of Day 1 (pre-reactivation, mean of final three trials) and the beginning of Day 3 (post reactivation, trial 1) for both the ketamine and placebo visits. Error bars represent SEM. *B. Sucking Pressure.* Anticipatory sucking to the cue predicting pleasant juice at the end of Day 1 (pre-reactivation) and the beginning of Day 3 (post-reactivation). Error bars represent SEM. *C. Expectancy Ratings.* Anticipatory liking ratings in response to the cue predicting pleasant juice at the end of Day 1 (pre-reactivation) and the beginning of Day 3 (post-reactivation). Error bars represent SEM.

We should consider the possibility that ketamine is having a primary and deleterious effect on extinction, a pathophysiological process implicated in spontaneous confabulation, another delusion-like phenomenon [99], [100], akin to the phenomenology of some ketamine experiences [12]. Indeed, extinction learning fails to consolidate in patients with schizophrenia [101]. While this is a reasonable consideration, a number of aspects of the experimental design and findings do not support this explanation (see Figure 9). First, day 1 entailed partial reinforcement and reactivation involved a brief single presentation of the conditioned stimulus. Both would shift the balance towards reconsolidation rather than extinction [19], [21]. Second, there was no sign of an extinction effect for the placebo reactivation condition and, if anything, the cue appeared to exert a stronger effect at the beginning of the post-reactivation session (day 3) than it did at the end of learning on the pre-reactivation session (day 1), see Figure 9. In short, a simple explanation of these findings in terms of an attenuation of extinction is unlikely.

Although schizophrenia is associated with amnesia [102], patients do report hypermnesia for their symptom contents [103], [104], and while these may be false memories [15], [105], they may be vivid enough to engender post traumatic stress disorder (PTSD) symptoms [106]. It is notable that such delusional false memories can occur following long-term anesthesia that blocks NMDA receptors [106]. The present data raise the intriguing possibility that patients with schizophrenia rely on non-NMDA mediated forms of synaptic plasticity to represent their world. These learning mechanisms may produce less veridical but more robust representations hence the bizarreness and persistence of psychosis.

There are important caveats to the inferences we draw from these data and clearly, they warrant various follow-up studies. To begin with, the imaging effects we report are of small magnitude and the sample sizes, particularly in study 2, are small. Furthermore, future studies should include a non-reactivated control condition, which we included in study 2 but not study 1. It will be important to characterize PE and reconsolidation in patients with fixed delusions since an important difference from ketamine is that, though the latter induces psychotic percepts and delusion-like ideas, most subjects retain insight (although not always [67]). It would also be useful in future studies to examine the relationship between appetitive conditioning to primary reward and our causal learning PE signal and important, too, to assay the degree of arousal subjects report in future appetitive studies, since this was the dimension modulated by ketamine and related to striatal PE signal. Preclinical studies are underway in order to replicate and interrogate this strengthening effect of ketamine and its possible synaptic mechanisms. A more invasive approach will permit investigation at the synaptic level and circumvent the necessarily correlational approach adopted presently. Of course, correlation is not causation but it is a crucial first step in demonstrating that processes (in this case, PE, memory reconsolidation and delusions) are related [107] through shared variation with individual differences in neural PE signal [108].

Overall, while we can only speculate on the precise underlying mechanisms, the key observation here is that the same manipulation that can render an individual vulnerable to delusion-like ideas may also lead to memory persistence. These data are consistent with a theoretical model of delusions in which aberrations of a fundamental learning process, PE, can profoundly and persistently affect an individual's world view. Thus data thus provide an empirical basis for a mechanistic understanding of what has for a long time been considered ‘*ununderstandable*’ [34]: the delusions that attend serious mental illnesses like schizophrenia. They demonstrate, moreover, the potential insights that clinical psychiatry may gain from developments in neuroscience.
